# Pyridyl group design in viologens for anolyte materials in organic redox flow batteries†

**DOI:** 10.1039/c8ra02641f

**Published:** 2018-05-22

**Authors:** Chen Chen, Shun Zhang, Yingzhong Zhu, Yumin Qian, Zhihui Niu, Jing Ye, Yu Zhao, Xiaohong Zhang

**Affiliations:** Institute of Functional Nano & Soft Materials (FUNSOM), Jiangsu Key Laboratory for Carbon-Based Functional Materials & Devices, Collaborative Innovation Centre of Suzhou Nano Science and Technology (Nano-CIC), Soochow University 199 Renai Road, Suzhou Industrial Park Suzhou Jiangsu 215123 P. R. China yuzhao@suda.edu.cn xiaohong_zhang@suda.edu.cn; Analytical and Testing Centre, Soochow University 199 Renai Road, Suzhou Industrial Park Suzhou Jiangsu 215123 P. R. China jingye@suda.edu.cn

## Abstract

Organic redox compounds represent an emerging class of active materials for organic redox-flow batteries (RFBs), which are highly desirable for sustainable electrical energy storage. The structural diversity of organic redox compounds helps in tuning the electrochemical properties as compared to the case of their inorganic counterparts. However, the structural diversity makes the design and identification of redox-active organic materials difficult because it is challenging to achieve appropriate redox potential, solubility and stability together, which are the major concerns regarding the practical applicability of these materials to RFBs. Herein, we report the design, synthesis, and application of viologen molecules as anolyte materials for organic RFBs that are compatible with Li-ion electrolytes. Structural screening assisted by density functional theory (DFT) calculations suggests that the (CH_2_)_5_CH_3_-substituted viologen molecule exhibits reduction potential as low as 2.74 V *vs.* Li/Li^+^, good structural stability due to effective charge delocalization within the two pyridinium rings, and a solubility of up to 1.3 M in carbonate-based electrolytes. When paired with a 2,2′:6′,2′′-terpyridine–iron complex catholyte, the cell shows a high discharge voltage of 1.3–1.5 V with coulombic efficiency > 98% and energy efficiency > 84%. Both the anolyte and catholyte materials are built from earth-abundant elements and can be produced with high yields; thus, they may represent a promising choice for sustainable electrical energy storage.

## Abstract 2

Organic redox compounds represent an emerging class of active materials for organic redox-flow batteries (RFBs), which are highly desirable for sustainable electrical energy storage.

## Graphical abstract

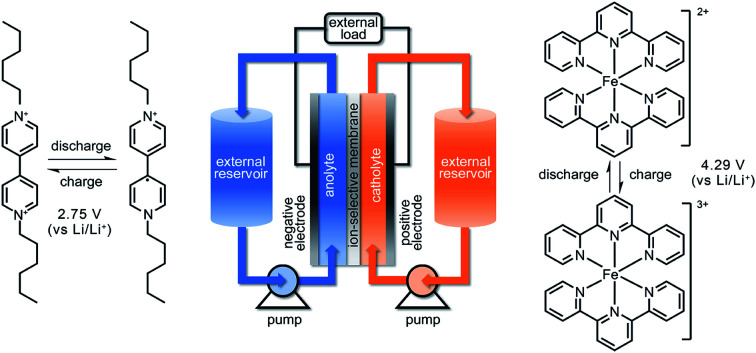

## Introduction

Sustainable electric energy storage systems are important as they enable full utilization of renewable electricity generated from intermittent energy sources such as wind and sun; redox flow batteries (RFBs) have been considered as one of the most important electric energy storage systems for upcoming large-scale applications.^1^ The unique feature of an RFB is the decoupling of power and energy; the former is controlled by the stack, whereas the latter is stored within the separated reactants. The distinctive cell architecture of RFBs provides a number of attractive merits such as satisfying the requirements of durability and calendar life, rapid response to changes in load or input, and affordable capital costs.^2^ Present RFBs are dominated by inorganic materials.^3^ For the upcoming large-scale applications, it is crucial to emphasize benign environmental attributes realized using elementally abundant active materials and material sustainability achieved using materials made through eco-efficient processes.^4^ Recent studies have demonstrated the promise of using redox-active organic materials as viable alternatives towards a new generation of RFBs;^5^ the merits of using organic materials arise from the structural diversity and tunability of electrochemical properties in addition to material sustainability and abundance.

In principle, an organic RFB can operate using either an aqueous or a non-aqueous electrolyte. The benefits of aqueous electrolytes include low solvent costs and high ionic conductivities, but aqueous electrolytes have limited charging potential due to water electrolysis; however, although non-aqueous electrolytes exhibit lower ionic conductivities and have higher capital costs, they expand the potential window; thus, they may increase the number of redox-active materials, especially in the negative electrode.^6^ In previous studies, many catholyte systems, either aqueous or non-aqueous, have been demonstrated by employing molecules such as quinones,^7^ N-oxides,^8^ arylamines,^9^ alkylarenes,^10^ thiophenes,^11^ thiadiazoles^12^ and others.^13^ Compared with aqueous anolyte systems, in which viologens^14^ and quinones^8*e*,15^ are employed, non-aqueous anolyte systems that are compatible with Li-ion electrolytes are rare and currently remain limited to insoluble lithium salts of aryl carboxylates,^16^ pyridine-based materials,^17^ quinoxalines,^18^ and thiadiazoles.^19^ The realization of practical non-aqueous organic RFBs strongly depends on the development of redox-active materials for the anolyte.^20^ The lower limit of the electrochemical window of non-aqueous electrolytes is substantially broader than that of the aqueous electrolyte, and an improvement in anolyte potential represents a substantial increase in energy density;^21^ to increase the possibilities of non-aqueous organic RFBs, it is essential to develop soluble organic anolyte materials, which require ingenious synthetic work as well as theoretical identification addressing the redox potential, solubility, and stability.^22^

Herein, we report the design, synthesis, and application of a (CH_2_)_5_CH_3_-substituted viologen molecule as an anolyte material for organic RFBs that is compatible with Li-ion electrolytes. Viologens possess unique and interesting properties including three stable redox states and tunability of the nitrogen substituents.^23^ Moreover, viologen radical cations are one of the most stable organic radicals, and their dicationic species can be prepared as air-stable salts.^24^ DFT calculations have been used to gain in-depth information on the redox potential, solubility and stability of viologen derivatives with various substituent groups on the N atoms in the pyridine/pyridinium rings. The (CH_2_)_5_CH_3_-substituted viologen molecule has been synthesized to serve as an anolyte material, which shows good structural stability, reduction potential as low as 2.74 V *vs.* Li/Li^+^, and a solubility of up to 1.3 M in carbonate-based solvents. When paired with 2,2′:6′,2′′-terpyridine–iron complex catholyte, the cell shows a discharge voltage of 1.3–1.5 V with coulombic efficiency > 98% and energy efficiency > 84%. Although a higher voltage is expected for non-aqueous organic RFBs, the redox potential and solubility of (CH_2_)_5_CH_3_-substituted viologen are comparable to the values that have been experimentally determined in non-aqueous organic RFB anolytes. The presented results highlight the great promise of the proposed viologen to achieve sustainability and producibility of organic RFBs for energy storage applications.

## Experimental

### Materials

All chemicals and solvents were used as received without further purification. Titanium foil (99.5%, 100 μm in thickness), copper foil (99.9%, 30 μm in thickness), and copper mesh were purchased from Nilaco Corporation. Iron(ii) chloride tetrahydrate (FeCl_2_·4H_2_O, 98%), ammonium tetrafluoroborate (NH_4_BF_4_, 97%), 4,4′-bipyridine, 2,2′:6′,2′′-terpyridine (98%), 1-iodohexane, bis(trifluoromethane) sulfonimide lithium salt (LiTFSI, 99.95%), and lithium metal were purchased from Sigma-Aldrich. The Li^+^-ion solid electrolyte was purchased from Ohara Corporation. Ethylene carbonate (EC, water content < 30 ppm) and dimethyl carbonate (DMC, water content < 30 ppm) were obtained from Kishida Chemicals. The polyvinylidene fluoride (PVDF) binder was obtained from MTI Corporation. *N*-Methyl-2-pyrrolidone (NMP) was obtained from J&K Chemicals. Super P carbon was obtained from TIMCAL.

### Synthesis of 1,1′-dihexyl-4,4′-bipyridinium tetrafluoroborate

4,4′-Bipyridine (1.56 g, 9.99 mmol), 1-iodohexane (4.24 g, 20.0 mmol), and 15 mL acetonitrile were placed in a high-pressure reactor. The reaction mixture was heated to 120 °C for 48 h. After filtration and washing with 5 mL acetonitrile for 3 times, the precipitate was added to an anion-exchange resin to change the anion from iodine to chloride. This was followed by concentration with percolate and treatment with a 5 mL saturated NH_4_BF_4_ aqueous solution to obtain a light-yellow solid. The pure product was dried under high vacuum. Light yellow solid, 4.75 g. Yield: 95%. ^1^H NMR (600 MHz, *d*_6_-DMSO) *δ* 9.40 (d, *J* = 6.9 Hz, 4H), 8.79 (d, *J* = 6.8 Hz, 4H), 4.68 (t, *J* = 7.5 Hz, 4H), 2.01–1.88 (m, 4H), 1.26 (dd, *J* = 14.8, 6.4 Hz, 13H), 0.84 (t, *J* = 7.0 Hz, 6H). ^13^C NMR (151 MHz, *d*_6_-DMSO) *δ* 149.20, 146.15, 127.09, 61.44, 31.14, 31.03, 25.53, 22.32, 14.27. MALDI-TOF MS (cal. 326.27, found: 326.26). Anal. calcd for C_22_H_34_B_2_F_8_N_2_: C, 52.83; H, 6.85, N, 5.60. Found: C, 52.81; H, 6.84, N, 5.62.

### Synthesis of bis(2,2′:6′,2′′-terpyridine)iron(ii) tetrafluoroborate

2,2′:6′,2′′-Terpyridine (0.478 g, 2.05 mmol), ferrous chloride tetrahydrate (0.199 g, 1.00 mmol), and degassed methanol (100 mL) were added to a 250 mL round-bottom flask under a nitrogen atmosphere. The mixture was heated to reflux for 48 h under strong magnetic stirring. After reaction, the solvent was removed by a rotary evaporator. The residue was washed 3 times with 50 mL diethyl ether to obtain a red-black powder. The crude product was dissolved in 30 mL methanol and filtered to remove insoluble impurities. The filtrate was treated with a 5 mL saturated NH_4_BF_4_ aqueous solution, and a bright red-black crystal was formed. The purified product was obtained by filtration and dried under high vacuum. Red-black crystal, 0.661 g. Yield: 95%. ^1^H NMR (600 MHz, *d*_6_-DMSO) *δ* 9.22 (d, *J* = 8.1 Hz, 4H), 8.79 (dd, *J* = 15.3, 7.8 Hz, 6H), 7.96 (t, *J* = 7.5 Hz, 4H), 7.15 (t, *J* = 6.4 Hz, 4H), 7.11 (d, *J* = 5.2 Hz, 4H). ^13^C NMR (151 MHz, *d*_6_-DMSO) *δ* 160.07, 157.99, 153.10, 139.25, 138.56, 128.07, 124.42, 124.31. MALDI-TOF MS (cal. 522.12, found: 522.10). Anal. calcd for C_30_H_22_B_2_F_8_FeN_6_: C, 51.77; H,3.19, N, 12.07. Found: C, 51.76; H, 3.18, N, 12.09.

### Determination of the solubility of 1,1′-dihexyl-4,4′-bipyridinium tetrafluoroborate and 1,1′-dihexyl-4,4′-bipyridine

To determine the solubility of 1,1′-dihexyl-4,4′-bipyridinium tetrafluoroborate and 1,1′-dihexyl-4,4′-bipyridine in bare EC/DMC solvent, excess 1,1′-dihexyl-4,4′-bipyridinium tetrafluoroborate (*ca.* 1.6 g) and 1,1′-dihexyl-4,4′-bipyridine (*ca.* 0.2 g) were separately added to the EC/DMC solvent (*ca.* 2 mL). After stirring for sufficient time, the upper clear solution was transferred into a volumetric flask (1 mL) and vaporized in vacuum at room temperature. The remaining 1,1′-dihexyl-4,4′-bipyridinium tetrafluoroborate and 1,1′-dihexyl-4,4′-bipyridine were weighed to be 0.65 g and 0.065 g, respectively, corresponding to a molar concentration of *ca.* 1.3 M and 0.2 M, respectively. To determine the solubility of 1,1′-dihexyl-4,4′-bipyridinium tetrafluoroborate in the EC/DMC electrolyte containing 1 M LiTFSI, excess 1,1′-dihexyl-4,4′-bipyridinium tetrafluoroborate (*ca.* 1.6 g) was added to the EC/DMC electrolyte (2 mL). The undissolved solids were filtered, and the weight was found to be 0.59 g, indicating that the solubility of 1,1′-dihexyl-4,4′-bipyridinium tetrafluoroborate was *ca.* 1 M in the EC/DMC electrolyte.

### Cyclic voltammetry studies

Cyclic voltammetry studies were carried out using a three-electrode configuration with a polyetheretherketone-encased glassy carbon disk (3 mm in diameter), a platinum wire, and a Ag/AgCl electrode serving as the working, the counter, and the reference electrode, respectively. The electrolyte was composed of 2 mM (CH_2_)_5_CH_3_-substituted Viol^2+^, 2 mM Fe-tpy and 0.25 M LiTFSI in the EC/DMC solvent. Prior to testing, the working electrode was polished with 0.05 mm Al_2_O_3_ powder, rinsed with deionized H_2_O, and dried with N_2_. CV profiles were obtained using potentiostat (CHI850D, CH Instruments) under an Ar atmosphere. The scan rate was 10 mV s^−1^.

### RDE studies

RDE measurements were conducted using a rotating disk electrode (RRDE-3A, ALS Co. Ltd.) in a three-electrode configuration with a glassy-carbon disk (3 mm in diameter) as the working electrode, a platinum wire as the counter electrode, and a Ag/AgCl electrode as the reference electrode. RDE profiles were obtained at various rotating speeds with a fixed sweeping rate of 10 mV s^−1^. The rotation speeds for the anolyte were 400, 625, 900, 1225, 1600, 2025, 2500, 3025 and 3600 rpm, and the rotation speeds for the catholyte were 1600, 2025, 2500, 3025, 3600, 4225, 4900, 5625, and 6400 rpm. *η* used for the calculation of *k*_0_ was 5, 10, 15, 20, 25, 35 and 45 mV. All tests were carried out at room temperature. The anolyte and catholyte were bubbled with N_2_ before the test.

### Cell measurements

Both the half-cell and flow cell were composed of custom-made components. For individual evaluation of the performance of (CH_2_)_5_CH_3_-substituted Viol^2+^ and Fe-tpy, a half-cell was assembled with two quartz shells (1 mm thick, 8 mm in diameter) and a NASICON-type Li_1+*x*+3*z*_Al_*x*_(Ti, Ge)_2−*x*_Si_3*z*_P_3−*z*_O_12_ Li^+^-ion conducting membrane (LICGC® AG-01, Ohara Corp., Japan) sandwiched in between. The components were sealed together with Surlyn® resin (Solaronix Meltonix 1170-25). A Ti foil pre-casted with a Super P carbon/PVDF thin layer was used as the current collector for the cathode, and a piece of Li foil pressed onto the Cu foil was used as the anode. The electrolyte was injected into the quartz shell chamber through a small hole on the Ti or Cu foil. The electrolyte used at the anode was 1 M LiTFSI in EC/DMC, whereas the electrolyte used in the cathode was 0.25 M (CH_2_)_5_CH_3_-substituted Viol^2+^ or Fe-tpy in EC/DMC containing 1 M LiTFSI. For the flow cell test, the electrodes were two 50 × 50 mm graphite chambers with a flow channel, which were 1 mm in depth and 5 cm^2^ in area. A NASICON-type Li_1+*x*+3*z*_Al_*x*_(Ti, Ge)_2−*x*_Si_3*z*_P_3−*z*_O_12_ Li^+^-ion conducting membrane was sandwiched between two graphite chambers. A thermoplastic sealing film made of Surlyn® (Solaronix Meltonix 1170-25) was used to seal the graphite chamber and the Li^+^-ion conducting membrane. Moreover, two endplates, machined out of solid stainless-steel, were used as current collectors. The cell structure is schematically shown in Fig. S1, ESI.† On the negative side of the cell, 0.25 M (CH_2_)_5_CH_3_ substituted Viol^2+^ and 1 M LiTFSI in EC/DMC were used as the anolyte in the fully discharged state; on the positive side, 0.25 M Fe-tpy and 1 M LiTFSI in EC/DMC were used as the catholyte. Peristaltic pumps were used to circulate the 5 mL fluids at a rate of approximately 1 mL min^−1^. All measurements shown herein were conducted at room temperature.

### Computational methods

Geometry optimizations were performed without restriction using the B3LYP hybrid density functional^25^ implemented in the Gaussian 09 suite of programs.^26^ The 6-31+g(d,p) basis sets were adopted for C, H, N and O atoms.^27^ The program's default threshold values for self-consistency-field energy, total energy and force are adopted. The highest occupied molecular orbital (HOMO)/lowest unoccupied molecular orbital (LUMO) energy level is calculated using the M062x functional after structure optimization. EC and DMC are used as the solvents within the SMD. The static dielectric constant *ε* = 89.6 (EC) × 0.3 + 3.11 (DMC) × 0.7 = 29.057 is set for these two solvent configurations. The rest of the solvent parameters are based on a solvent that has a similar dielectric constant as EC : DMC = 3 : 7 (ε = 26.726, 2,2,2-trifluoroethanol). The thermal correction to Gibbs free energy is obtained from vibrational frequency calculations in the solvent model. No negative frequency is found in any of the optimized structures. The redox potential (*E*_red_^1/2^) is calculated using the formula *E*_red_^1/2^ = (*G*^0^_f_ − *G*^0^_i_)/*nF* − *C*, where *G*^0^_f_ and *G*^0^_i_ are the sum of electronic and thermal Gibbs free energy of the final and initial states of the organic molecule, respectively, *n* is the number of electrons in the redox process, *F* is the Faraday constant (23.061 kcal per volt-mol), and *C* is a constant, which is relevant to the reference electrode.

## Results & discussion

**Fig. 1:**
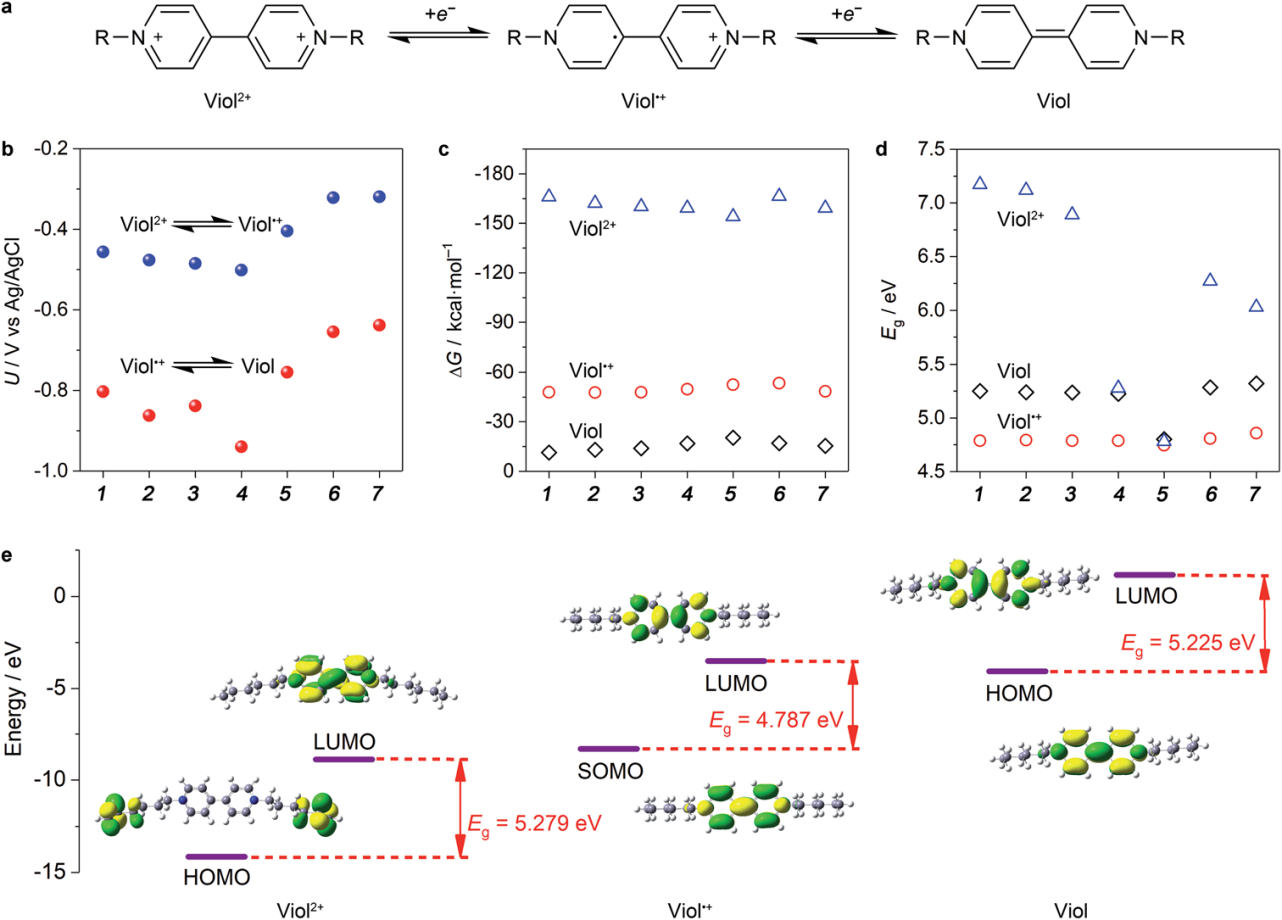
General redox mechanism and DFT screening of viologen derivatives. (a) Redox mechanism of two successive one-electron transfer reactions occurred at viologen derivatives. (b) Redox potential, (c) solvation free energy (Δ*G*) and (d) HOMO–LUMO energy gap of Viol, Viol˙^+^ and Viol^2+^ with seven substituted groups of –CH_3_ (1), –CH_2_CH_3_ (2), –(CH_2_)_2_CH_3_ (3), –(CH_2_)_5_CH_3_ (4), –CH_2_(C_6_H_5_) (5), –CH_2_COOCH_3_ (6) and –CH_2_OCH_3_ (7). (e) DFT-optimized structure and frontier HOMO/SOMO and LUMO orbitals of (CH_2_)_5_CH_3_-substituted Viol, Viol˙^+^ and Viol^2+^.

Among the bipyridine derivatives, viologen derivatives are of particular interest and have been utilized as an anolyte material in aqueous organic RFBs recently.^14^ These molecules (denoted as Viol, Viol˙^+^ and Viol^2+^, as shown in Fig. 1a) undergo two successive one-electron transfer reactions. The reversibility of electron transfer reactions, redox potential, solubility and energy gap (*E*_g_) of the HOMO–LUMO of viologen derivatives should be strongly related to the nitrogen substituents, which have been first screened by DFT calculations. For anolyte applications, the redox potential should be more negative such that to maximize the cell voltage. The calculated redox potential of viologen derivatives (Fig. 1b) revealed that when an alkyl chain was used as the substituent group, the redox potential gradually shifted towards lower potentials with an increase in the number of C atoms in the alkyl chains, in accordance with their enhanced electron donor effect. In contrast, when benzyl, methoxycarbonylmethyl and methoxymethyl were used as the substituent groups, the redox potential increased in accordance with the strength of the electron withdrawal effect. Hence, it is preferable to use electron-donating alkyl chains as substituent groups in viologen derivatives.

Another important aspect is solubility, which is in proportion to the energy capacity of the anolyte. The calculated solvation free energy of Viol, Viol˙^+^ and Viol^2+^ (Fig. 1c) suggested that the positively charged Viol˙^+^ and Viol^2+^ showed more negative solvation free energy as compared to the neutral species; this indicated better solubility. According to the frontier molecular orbitals theory, small gaps between energy levels contribute to the enhanced conductivity of a molecule, which allow the molecule to have a strong flow of electrons.^28^ Thus, it may consolidate the utilization ratio of redox-active molecules by means of electron transfer at the interface of the current collector. It is demonstrated that the order of the utilization ratio of redox-active molecules almost follows the same trend as that of the *E*_g_ values, whereby a smaller gap corresponds to a higher utilization ratio.^29^ For each viologen derivative, Viol˙^+^ exhibited smallest *E*_g_, which was due to the unpaired electron, followed by Viol and Viol^2+^ (Fig. 1d). Particularly, the *E*_g_ decreased as the number of C in the alkyl chain increased. (CH_2_)_5_CH_3_- and benzyl-substituted Viol showed smaller *E*_g_ than other substituent groups in Viol^2+^. However, the latter showed higher redox potential. It should be noted that the first redox reaction between Viol˙^+^ and Viol^2+^ is fully reversible, but the second redox reaction between Viol and Viol˙^+^ may lead to side reactions.^30^ Therefore, for anolyte application, it is practical to utilize the redox reaction between Viol˙^+^ and Viol^2+^ in (CH_2_)_5_CH_3_-substituted Viol while taking redox potential, solubility and *E*_g_ into consideration together.

The chemical and electronic structure of (CH_2_)_5_CH_3_-substituted viologen was further investigated to reveal the redox-active moieties and thermodynamic stability of (CH_2_)_5_CH_3_-substituted viologen in different valence states (Fig. 1e). For (CH_2_)_5_CH_3_-substituted Viol^2+^, the C–C bond length between two pyridinium rings was 1.487 Å, which could be assigned to a C–C single bond. The two pyridinium rings showed a dihedral angle of 41.89°. Both HOMO and LUMO were symmetrically spread throughout the two pyridinium rings. For (CH_2_)_5_CH_3_-substituted Viol˙^+^, the C–C bond length between two pyridine–pyridinium rings was 1.425 Å. The shortened C–C bond could be attributed to the π-bonding interaction, revealed by the SOMO (singly occupied molecular orbital) of (CH_2_)_5_CH_3_-substituted Viol˙^+^. The unpaired electron was highly delocalized within two pyridine–pyridinium rings. The two pyridine–pyridinium rings were coplanar, with a very small dihedral angle of 0.151°, a striking structural change as compared to (CH_2_)_5_CH_3_ substituted Viol^2+^. However, for (CH_2_)_5_CH_3_-substituted Viol, the C–C bond length between the two pyridine rings was 1.37 Å, which could be assigned to a C=C double bond in its resonance structure. Its HOMO also confirmed high charge delocalization within its two co-planar rings and an even smaller dihedral angle of 0.13°. According to the molecular orbital theory, charge delocalization is essential to stabilize the energetic Viol/Viol˙^+^/Viol^2+^ and echoes the electrochemical reversibility and stability. As indicated by the molecular orbital theory, the redox-active moieties were focused on the two pyridine and/or pyridinium rings. It should be noted that the electrons are rather focused on the alkyl group of Viol^2+^ instead of the two pyridinium rings. A plausible explanation might be the higher electronic energy of the alkyl group than that of the two pyridinium rings. A similar phenomenon has been found in many other substituted structures (Table S1, ESI†).

**Fig. 2:**
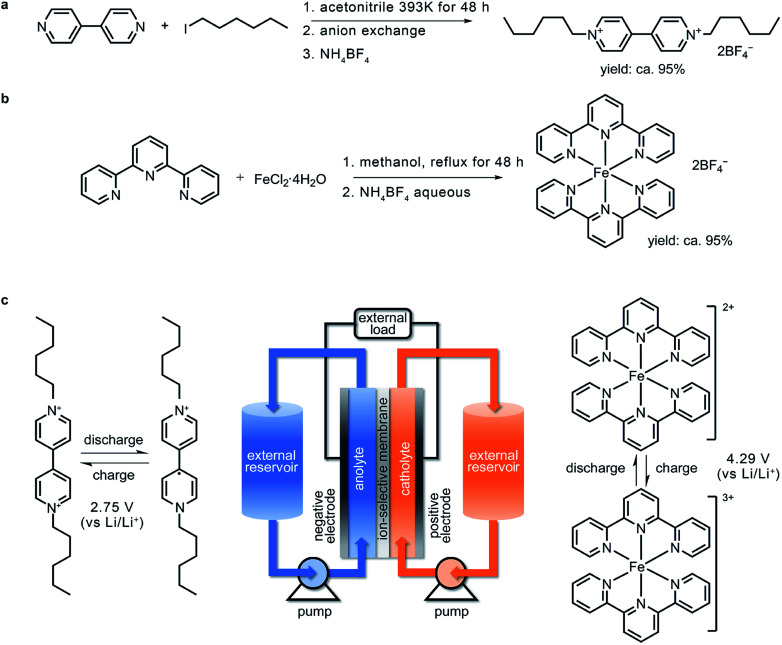
Material preparation and design of the (CH_2_)_5_CH_3_-substituted Viol^2+^|Fe-tpy RFB. The synthetic routes for (a) (CH_2_)_5_CH_3_-substituted Viol^2+^ and (b) Fe-tpy. (c) Schematic of the (CH_2_)_5_CH_3_-substituted Viol^2+^|Fe-tpy RFB with the corresponding cell reactions in the anolyte (left) and catholyte (right).

Based on the calculation results, (CH_2_)_5_CH_3_-substituted Viol^2+^ was synthesized *via* the solvothermal reaction of 4,4′-bipyridine and 1-iodohexane in a high-pressure reactor followed by soaking in an anion-exchange resin (Fig. 2a). To match this anolyte material, a high-voltage terpyridine-based Fe^2+^ complex (denoted as Fe-tpy) was synthesized (Fig. 2b) and used as the active material in the catholyte. In this iron complex, the lowest energy double-occupied Fe 3d orbital serves as the redox centre (Fig. S2, ESI†). The working principle and architecture of this organic RFB are presented in Fig. 2c, and the charging process can be expressed as eqn (1)–(3).

1 Negative electrode: Viol^2+^ + e^−^ → Viol˙^+^

2 Positive electrode: Fe(ii)-tpy − e^−^ → Fe(iii)-tpy

3 Overall: Viol^2+^ + Fe(ii)-tpy → Viol˙^+^ + Fe(iii)-tpy

**Fig. 3:**
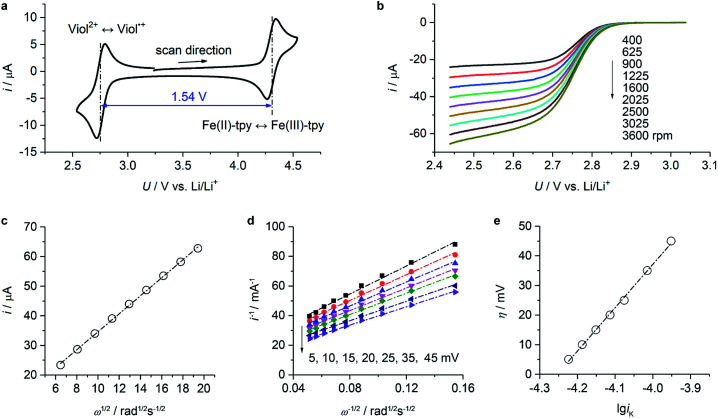
CV and RDE studies for (CH_2_)_5_CH_3_-substituted Viol^2+^. (a) CV profile of (CH_2_)_5_CH_3_-substituted Viol^2+^ and Fe(ii)-tpy mixed electrolyte in EC/DMC containing 0.25 M LiTFSI at a scan rate of 10 mV s^−1^. (b) RDE profiles of (CH_2_)_5_CH_3_-substituted Viol^2+^ in EC/DMC. The electrolyte was composed of 2 mM (CH_2_)_5_CH_3_-substituted Viol^2+^, 0.25 M LiTFSI in EC/DMC. The scan rate was 10 mV s^−1^. (c) Limiting current (*i*_lim_) *vs.* square root of rotation speed (*ω*^1/2^) derived from (b). (d) *η*-Dependent current as a function of *ω*^−1/2^ derived from (a). (e) *η* as a function of lg *i*_K_ upon the first reduction of (CH_2_)_5_CH_3_-substituted Viol^2+^. The *x*-intercept provides the log of *i*_0_.

Cyclic voltammetry data of (CH_2_)_5_CH_3_-substituted Viol^2+^ and Fe(ii)-tpy (Fig. 3a) exhibited well-defined oxidation and reduction peaks with a half-wave potential of 2.75 V *vs.* Li/Li^+^ for Viol^2+^ and 4.29 V *vs.* Li/Li^+^ for Fe(ii)-tpy. The former was very close to the calculated value of 2.74 V *vs.* Li/Li^+^ (Fig. 1b). The two sets of redox potentials observed for (CH_2_)_5_CH_3_-substituted Viol^2+^ and Fe(ii)-tpy were about 1.54 V apart, suggesting that a cell voltage of 1.5 V could be achieved by pairing (CH_2_)_5_CH_3_ substituted Viol^2+^ anolyte with the Fe(ii)-tpy catholyte. To confirm the qualification of these compounds as active materials in the proposed organic RFB, the diffusion coefficient and redox kinetics were investigated by rotating disk electrode (RDE) studies. Fig. 3b and S3 (ESI†) show the RDE profiles of (CH_2_)_5_CH_3_ substituted Viol^2+^ anolyte and Fe(ii)-tpy catholyte, respectively. The diffusion coefficient (*D*) is determined by Levich plots (Fig. 3c and S4, ESI†), which is defined as *i*_lim_ = 0.620 × *nFD*^2/3^*Aω*^1/2^*ν*^−1/6^*c*, where *i*_lim_ is the limiting current, *n* is the number of electrons transferred during the redox reaction, *F* is the Faraday constant (96 485 C mol^−1^), *A* is the surface area of the rotating disk (0.0707 cm^2^), *ω* is the rotation speed of the working electrode, *ν* is the kinematic viscosity of the solution (3.2 × 10^−2^ cm^2^ s^−1^), and *c* is the bulk concentration of the active materials (2 × 10^−6^ mol cm^−3^). The diffusion coefficient was calculated to be 3.0 × 10^−6^ cm^2^ s^−1^ for (CH_2_)_5_CH_3_-substituted Viol^2+^ and 4.1 × 10^−6^ cm^2^ s^−1^ for Fe(ii)-tpy. The reciprocal of the current at various overpotentials (*η*) was plotted *versus ω*^−1/2^ (Fig. 3d and S5, ESI†). The data for each potential were fitted with a straight line; the intercept provided the reciprocal of the kinetic current (*i*_K_, the extrapolation to infinite rotation rate). The *x*-intercept of the fitted overpotential *versus* lg *i*_K_ provided the log of the exchange current (*i*_0_, Fig. 3e and S6, ESI†), which was equal to *nFk*_0_*C*, where *k*_0_ is the electron-transfer rate constant. The calculated electron-transfer rate constant was 4.1 × 10^−3^ cm s^−1^ for (CH_2_)_5_CH_3_-substituted Viol^2+^ and 2.2 × 10^−3^ cm s^−1^ for Fe(ii)-tpy. Both (CH_2_)_5_CH_3_-substituted Viol^2+^ and Fe(ii)-tpy exhibit comparable diffusion coefficients and rate constants with other organic compounds applied in organic RFBs.^6,31^ The electrochemical properties of (CH_2_)_5_CH_3_-substituted Viol^2+^ and Fe-tpy are summarized in Table 1.

**Table 1:** A summary of the electrochemical properties of (CH_2_)_5_CH_3_-substituted Viol^2+^ and Fe-tpy

Compound	Redox potential (V *vs.* Li/Li^+^)	Peak separation (mV)	Peak current ratio^a^	*D* (cm^2^ s^−1^)	*k*_0_ (cm s^−1^)
(CH_2_)_5_CH_3_-substituted Viol^2+^	2.75	69	0.78	3.0 × 10^−6^	4.1 × 10^−3^
Fe(ii)-tpy	4.29	72	1.22	4.1 × 10^−6^	2.2 × 10^−3^

a Peak cathodic current/peak anodic current.

**Fig. 4:**
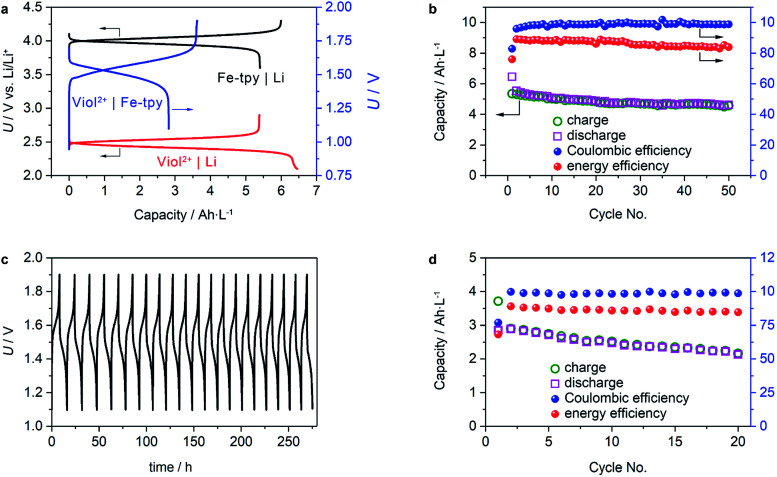
Cell performance. (a) Initial galvanostatic charge/discharge profiles of (CH_2_)_5_CH_3_-substituted Viol^2+^|Li, Fe-tpy|Li half-cells, and (CH_2_)_5_CH_3_-substituted Viol^2+^|Fe-tpy cell. (b) Cycling performance of (CH_2_)_5_CH_3_-substituted Viol^2+^|Li half-cell. (c) Galvanostatic charge/discharge profiles and (d) cycling performance of (CH_2_)_5_CH_3_-substituted Viol^2+^|Fe-tpy cell. In all the measurements, the concentration of anolyte and catholyte is 0.25 M, and the applied current density is 0.5 mA cm^−2^. The capacity is calculated based on *C* = *Nc*_a_*F*/*n*, where *N* is the number of electrons involved in the redox reaction (*N* = 1 in this case), *c*_a_ is the concentration of active redox species, *F* is the Faraday constant (26.8 A h mol^−1^), and *n* is the number of electrolyte volumes contributing to redox reactions (*n* = 2 in this case). The theoretical capacity for (CH_2_)_5_CH_3_-substituted Viol^2+^|Li and Fe-tpy|Li cells is 6.7 A h L^−1^ (based on the total volume of the anolyte or catholyte), while the theoretical capacity for (CH_2_)_5_CH_3_-substituted Viol^2+^|Fe-tpy cell is 3.35 A h L^−1^ (based on the total volume of the anolyte and catholyte).

Regarding the physical and chemical properties of (CH_2_)_5_CH_3_-substituted Viol^2+^ and Fe-tpy, an anion exchange mechanism should be applicable for performance evaluation. However, crossover is a known issue in polymeric cationic or anionic conducting membranes. Instead, we used a NASICON-type Li_1+*x*+3*z*_Al_*x*_(Ti, Ge)_2−*x*_Si_3*z*_P_3−*z*_O_12_ Li^+^-ion conducting membrane to evaluate the cell performance. Galvanostatic charge/discharge tests of half-cells using (CH_2_)_5_CH_3_-substituted Viol^2+^ or Fe(ii)-tpy as the cathode and Li metal as the anode demonstrated a stable voltage plateau and a relatively high utilization ratio of the active materials. The (CH_2_)_5_CH_3_-substituted Viol^2+^|Li half-cell showed a voltage plateau of 2.45–2.3 V (*vs.* Li/Li^+^) during discharge and 2.5–2.6 V (*vs.* Li/Li^+^) during charge (Fig. 4a). Moreover, the Fe-tpy|Li half-cell showed a voltage plateau of 3.95–4.15 V (*vs.* Li/Li^+^) during charge and 4.05–3.85 V (*vs.* Li/Li^+^) during discharge. These characteristics of the half-cells resulted in a stable voltage plateau for the (CH_2_)_5_CH_3_-substituted Viol^2+^|Fe-tpy cell, and the charging and discharging processes occurred within a narrow overpotential of 0.2 V. The active material utilization ratio of the initial cycle was 84% and 90% for (CH_2_)_5_CH_3_-substituted Viol^2+^|Li half-cell and Fe-tpy|Li half-cell, respectively, and 78% for the (CH_2_)_5_CH_3_-substituted Viol^2+^|Fe-tpy cell. The concentration polarization, arising from limited transport capabilities of both the separator and electrolyte diluent, became more obvious near the end of the charge/discharge process. Since both redox reactions exhibit relatively high activity and rapid electron-transfer kinetics, it is expected that the utilization ratio of active materials can be further enhanced by optimization of the electrode architecture to shorten the diffusion length of active materials. The half-cells also exhibited stable cycling performance. (CH_2_)_5_CH_3_-substituted Viol^2+^|Li half-cell demonstrated a capacity retention of ∼86% (based on the charge capacity), coulombic efficiency ∼99%, and energy efficiency of ∼87% in the measured 50 cycles (Fig. 4b). The corresponding values for Fe-tpy|Li half-cell were ∼100% (based on the discharge capacity), ∼99.5% and ∼90% (Fig. S7, ESI†).

To further validate the performance of a full cell, cycling performance of the (CH_2_)_5_CH_3_-substituted Viol^2+^|Fe-tpy RFB was examined. The investigated cell delivered stable capacity retention when tested over extended cycles. The charge/discharge voltage profiles over time revealed that the charge/discharge potential remained stable: about 1.5–1.7 V during charging and 1.5–1.3 V during discharging (Fig. 4c). The capacity retention was about 80% after 20 cycles. It should be mentioned that the capacity loss of (CH_2_)_5_CH_3_-substituted Viol^2+^ should result from the Li^+^-ion conducting membrane by means of a gradual reduction of Ti^IV^, which occurs when the potential threshold is below 2.8 V (*vs.* Li/Li^+^).^32^ On average, the coulombic efficiency and energy efficiency were *ca.* 98.2% and 84.8%, respectively, for a single charge/discharge cycle. The cell could reversibly deliver an energy density of *ca.* 4 W h L^−1^, which is comparable to that of the existing anolytes based on viologen derivatives (Table S2, ESI†).^8*e*,8*g*,14*b*–*d*^ In this study, (CH_2_)_5_CH_3_-substituted Viol^2+^ exhibited a high solubility: *ca.* 1.3 M in EC/DMC and *ca.* 1 M in EC/DMC containing 1 M LiTFSI (see the experimental section for more details). It should be mentioned that Fe-tpy only shows a solubility of 0.3 M, which is the bottleneck for achieving high energy density. Moreover, the low solubility of neutrally charged viologen derivatives prevent them from being used in concentrated non-aqueous anolytes although second electron transfer of CH_3_-substituted Viol^2+^ has been accessed recently.^33^

## Conclusions

In conclusion, the present study highlights the investigation of functionalized 4,4-bipyridine as anolytes for non-aqueous organic RFBs. The effects of different kinds of substituted groups have been investigated by DFT calculations, which regulate the electrochemical characteristics with molecular structures. Functionalization of 4,4-bipyridine with alkyl chains offers the possibility to develop stable anolytes with low reduction potentials and high solubilities. Viable electrochemical performance has been achieved upon pairing an anolyte of (CH_2_)_5_CH_3_-substituted 4,4-bipyridine with a catholyte based on an iron complex. The overall cell performance can be further boosted by proper molecular engineering with emphasis on the synthesis that uses safe and abundant organic raw materials.

## Conflicts of interest

There are no conflicts to declare.

## Supplementary Material

RA-008-C8RA02641F-s001
