# Supplementary material for: Pyridyl group design in viologens for anolyte materials in organic redox flow batteries
Source: RSC Adv. 2018 May 22;8(34):18762–70. doi: 10.1039/c8ra02641f (PMC9080602; doi:10.1039/c8ra02641f)
Supplement: RA-008-C8RA02641F-s001 [file RA-008-C8RA02641F-s001.pdf]

Electronic Supplementary Material (ESI) for RSC Advances.
This journal is © The Royal Society of Chemistry 2018

Electronic Supplementary Information (ESI) for

# The pyridyl group design in viologens for anolyte materials in organic redox flow batteries †

Chen Chen, ‡[a] Shun Zhang, ‡[a] Yingzhong Zhu,[a] Yumin Qian,[a] Zhihui Niu,[a] Jing Ye,*[b] Yu Zhao*[a] and Xiaohong Zhang*[a]

[a] Institute of Functional Nano & Soft Materials (FUNSOM), Jiangsu Key Laboratory for Carbon-Based Functional Materials & Devices, Collaborative Innovation Centre of Suzhou Nano Science and Technology (Nano-CIC), Soochow University, 199 Renai Road, Suzhou Industrial Park, Suzhou, Jiangsu 215123, P. R. China. Email: yuzhao@suda.edu.cn, xiaohong_zhang@suda.edu.cn.

[b] Analytical and Testing Centre, Soochow University, 199 Renai Road, Suzhou Industrial Park, Suzhou, Jiangsu 215123, P. R. China. Email: jingye@suda.edu.cn.

‡ These authors contributed equally to this work.

Table S1. The molecular orbital of Viol, Viol$^{\cdot+}$ and Viol$^{2+}$ with different substituted groups.

| Molecular structure | Molecular orbital | Viol | Viol$^{\cdot+}$ | Viol$^{2+}$ |
|---|---|---|---|---|
| 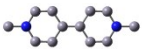<br>*1* | HOMO/ SOMO<br>LUMO | 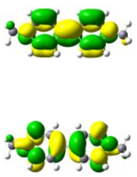 | 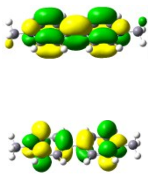 | 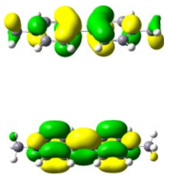 |
| 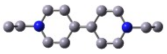<br>*2* | HOMO/ SOMO<br>LUMO | 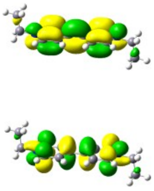 | 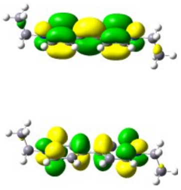 | 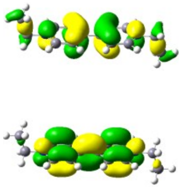 |
| 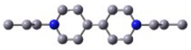<br>*3* | HOMO/ SOMO<br>LUMO | 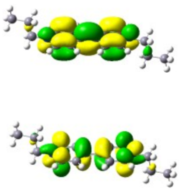 | 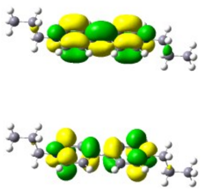 | 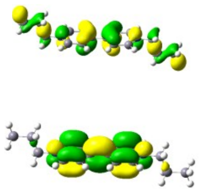 |
| 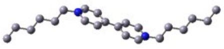<br>*4* | HOMO/ SOMO<br>LUMO | 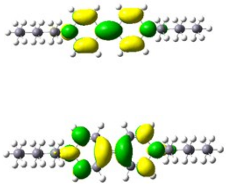 | 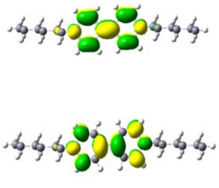 | 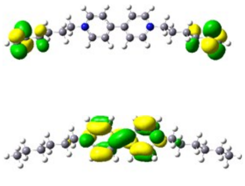 |
| 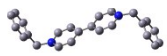<br>*5* | HOMO/ SOMO<br>LUMO | 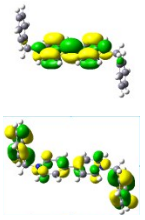 | 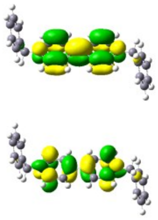 | 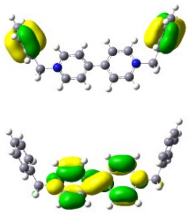 |
| 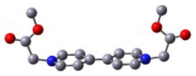<br>*6* | HOMO/ SOMO<br>LUMO | 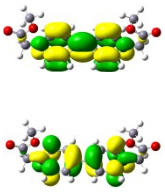 | 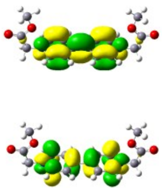 | 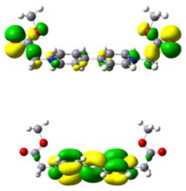 |
| 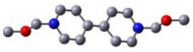<br>*7* | HOMO/ SOMO<br>LUMO | 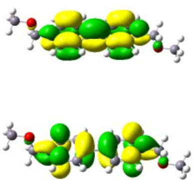 | 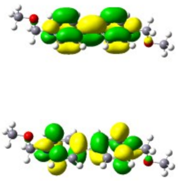 | 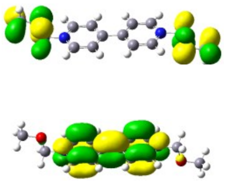 |

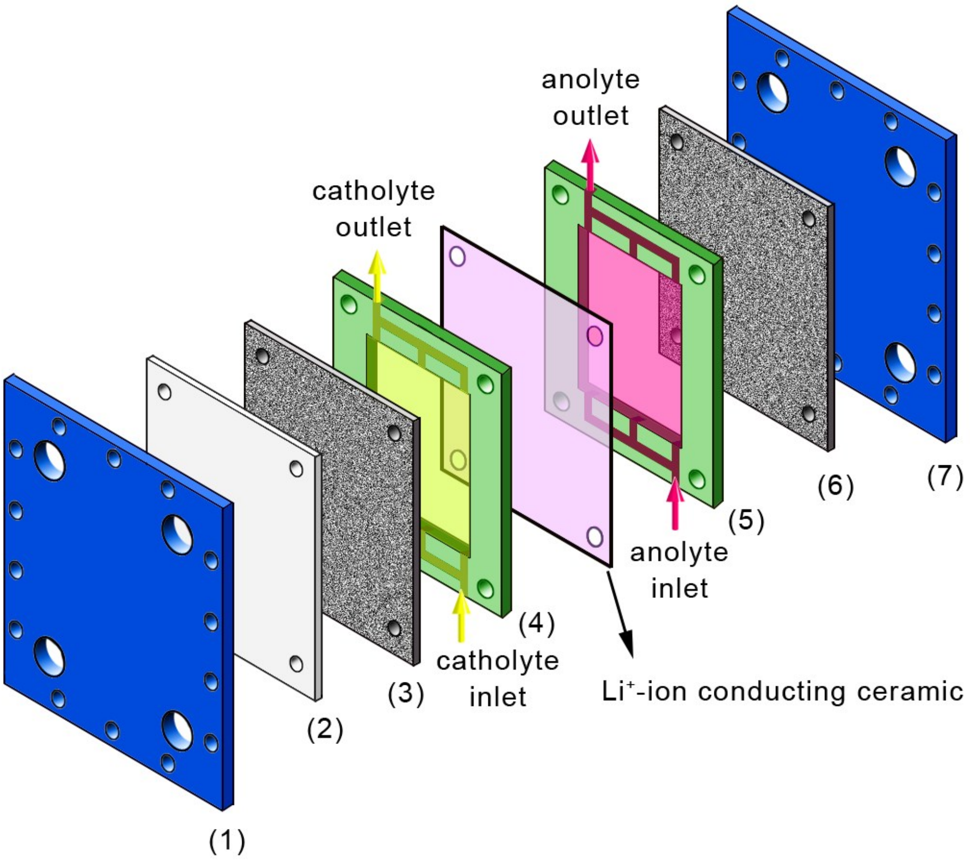


Cathode compartment:
(1) stainless-steel endplate
(2) isolation plate
(3) graphite plate
(4) graphite chamber

Anode compartment:
(5) graphite chamber
(6) graphite plate
(7) stainless-steel endplate

**Fig. S1** Schematic demonstration of the cell structure.

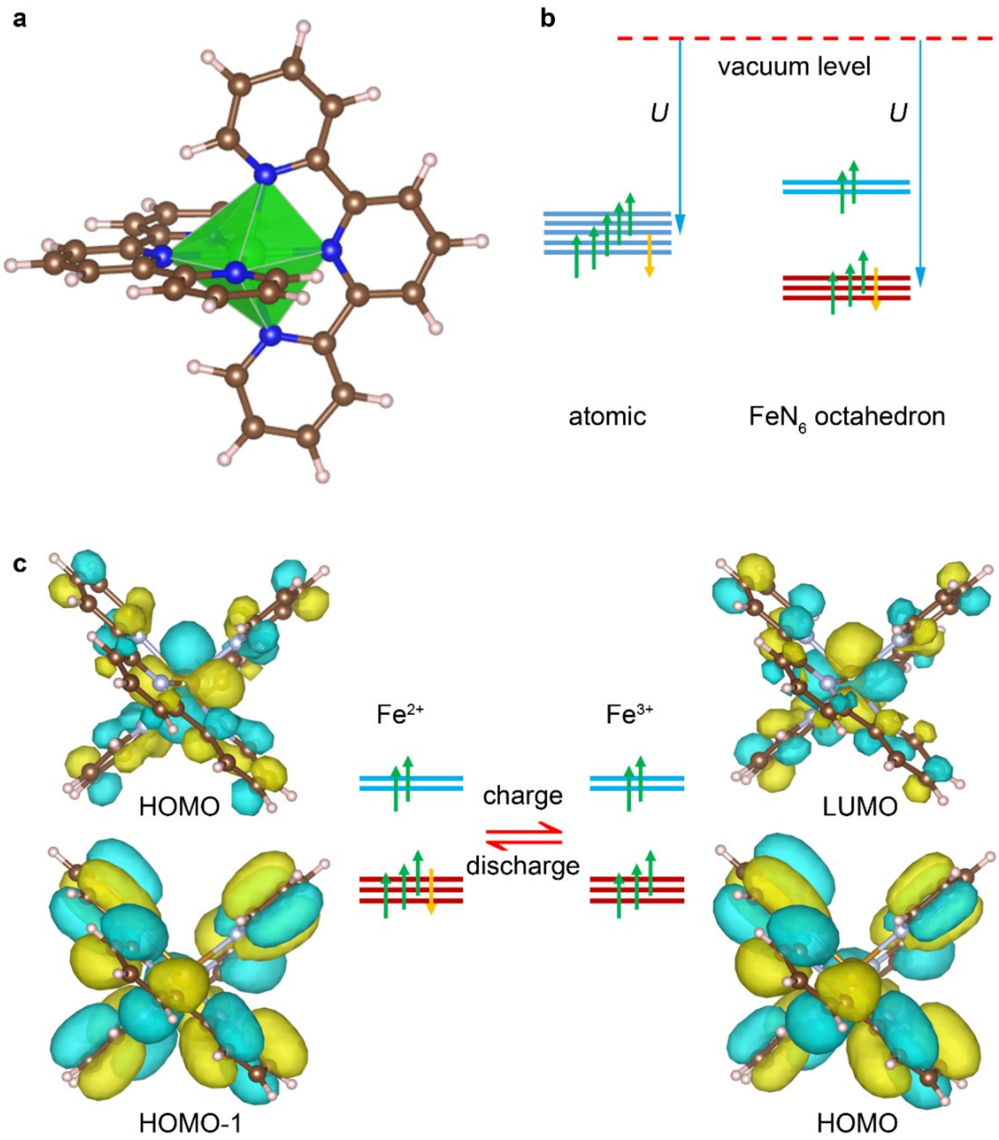


**Fig. S2** (a) Optimized the geometry of Fe-tpy molecule. (b) the splitting of five Fe 3*d* orbital and corresponding electronic configuration. (c) Spin differentiated LUMO-HOMO of the Fe-tpy and charging and discharging states. Electron configurations of all the molecules were calculated by DFT method within the framework of the Gaussian 09 package. [S1] The standard Pople basis set, 6-31G++(d,p), combined with the Lee–Yang–Parr exchange correlation functional (B3LYP) was used for all calculations. [S2] For each molecule, the geometry was fully optimized to achieve the lowest total energy before energy level calculation, and all possible spin multiplicities were explored (S=3,4,5,6), among which we chose the one with the lowest energy for analysis and comparison between different molecules.

The calculation shows the Fe ion is coordinated by the six nitrogen atoms from the two perpendicularly arranged tpy ligands. The $FeN_6$ forms a distorted octahedron, the average bond length of the $FN_6$ is from 2.18-2.25 A, as shown in Fig S1a. The five 3*d* orbital of Fe ions is split into two group: $e_g$ and $t_{2g}$. The energy scale of such splitting of the orbital group is 0.8 eV which is smaller than the usual Hund's coupling strength. The six electrons occupying the five 3*d* orbital

will form the high spin structure. According to the Hund's rule, all the orbital will be occupied with the lowest energy orbital double occupied, as shown in Fig. S1b. During the charging/discharging process the double occupied orbital will lose and gain electron due to the Hund's coupling rule to maintain the thermal dynamic minimum of electrons. Therefore, the lowest energy double occupied 3*d* orbital will be serve as the redox active center, which increases the redox potential compared to $Fe^{2+}/F^{3+}$ redox potential. To further confirm this point, the spin differentiated LUMO-HOMO of the Fe-tpy and charging and discharging state is explored. As show in Fig. S1c, the HOMO of the $Fe^{2+}$ is highly resemble to the LUMO of the $Fe^{3+}$. It is clear this orbital serves as redox active center during the charge and discharge process.

Reference:

[S1] Gaussian 09, Revision D.01, M. J. Frisch, G. W. Trucks, H. B. Schlegel, G. E. Scuseria, M. A. Robb, J. R. Cheeseman, G. Scalmani, V. Barone, B. Mennucci, G. A. Petersson, H. Nakatsuji, M. Caricato, X. Li, H. P. Hratchian, A. F. Izmaylov, J. Bloino, G. Zheng, J. L. Sonnenberg, M. Hada, M. Ehara, K. Toyota, R. Fukuda, J. Hasegawa, M. Ishida, T. Nakajima, Y. Honda, O. Kitao, H. Nakai, T. Vreven, J. A. Montgomery, Jr., J. E. Peralta, F. Ogliaro, M. Bearpark, J. J. Heyd, E. Brothers, K. N. Kudin, V. N. Staroverov, R. Kobayashi, J. Normand, K. Raghavachari, A. Rendell, J. C. Burant, S. S. Iyengar, J. Tomasi, M. Cossi, N. Rega, J. M. Millam, M. Klene, J. E. Knox, J. B. Cross, V. Bakken, C. Adamo, J. Jaramillo, R. Gomperts, R. E. Stratmann, O. Yazyev, A. J. Austin, R. Cammi, C. Pomelli, J. W. Ochterski, R. L. Martin, K. Morokuma, V. G. Zakrzewski, G. A. Voth, P. Salvador, J. J. Dannenberg, S. Dapprich, A. D. Daniels, Ö. Farkas, J. B. Foresman, J. V. Ortiz, J. Cioslowski, and D. J. Fox, Gaussian, Inc., Wallingford CT, 2009

[S2] A. D. Becke, *J. Chem. Phys.*, 1993, **98**, 5648.

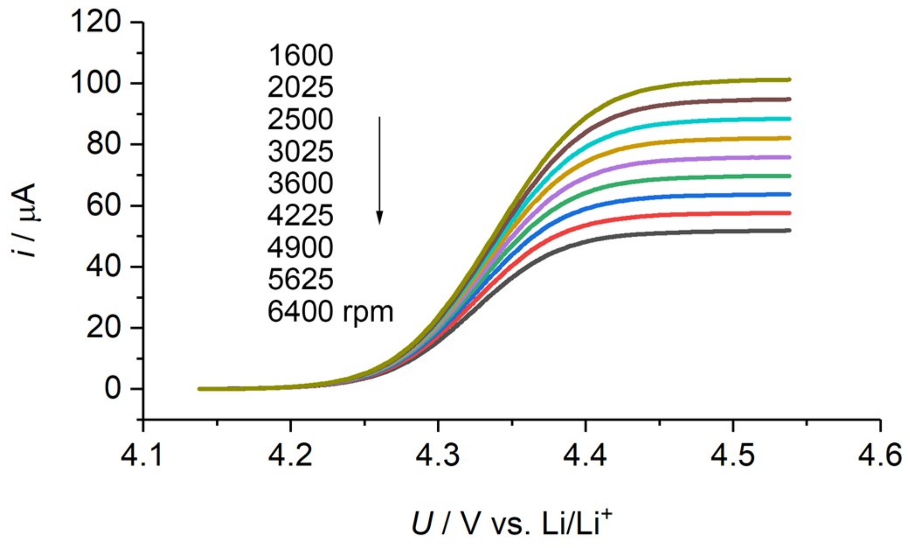


**Fig. S3** RDE profiles of Fe(II)-tpy in EC/DMC. The electrolyte was composed of 2 mM Fe(II)-tpy, 0.25 M LiTFSI in EC/DMC. The scan rate was 10 mV·s$^{-1}$.

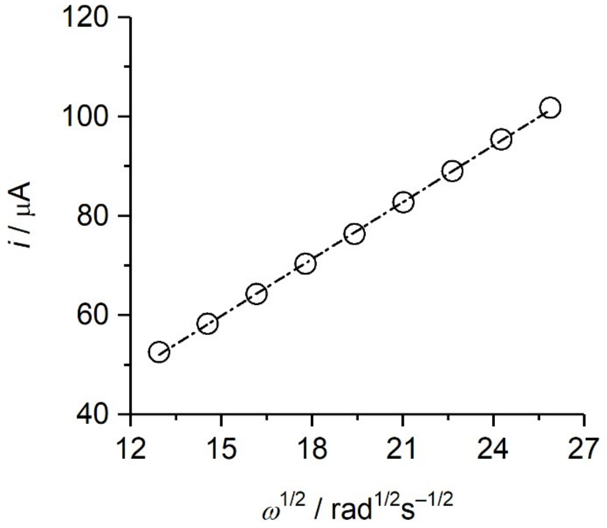


**Fig. S4** Limiting current ($i_{\mathrm{lim}}$) vs. square root of rotation speed ($\omega^{1/2}$) derived from Fig. S3.

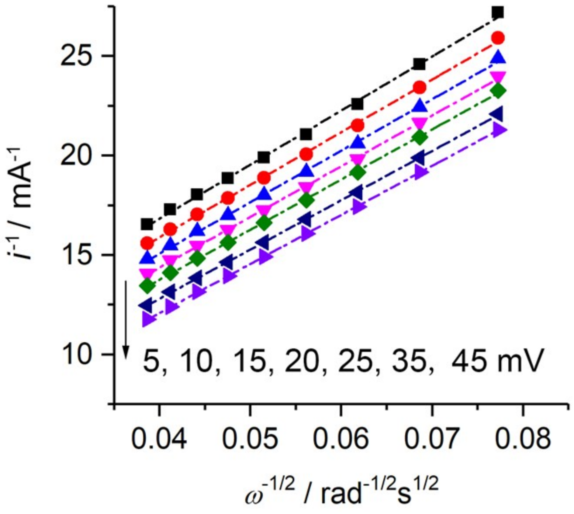


**Fig. S5** $\eta$-dependent current as a function of $\omega^{-1/2}$ derived from Fig. S3.

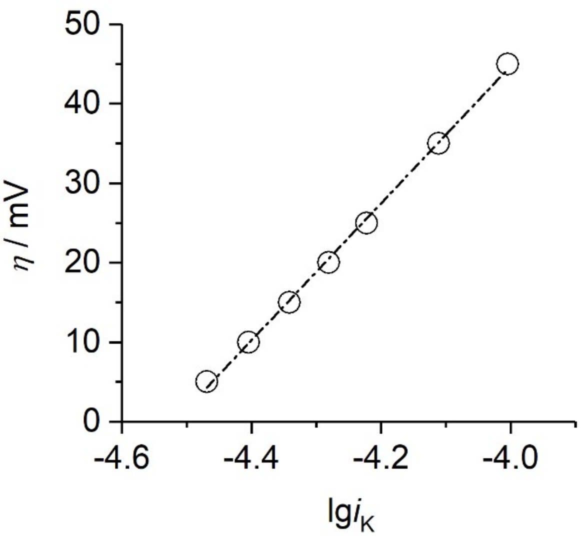


**Fig. S6** $\eta$ as a function of lg$i_K$ upon the oxidation of Fe(II)-tpy. The $x$-intercept gives the log of $i_0$.

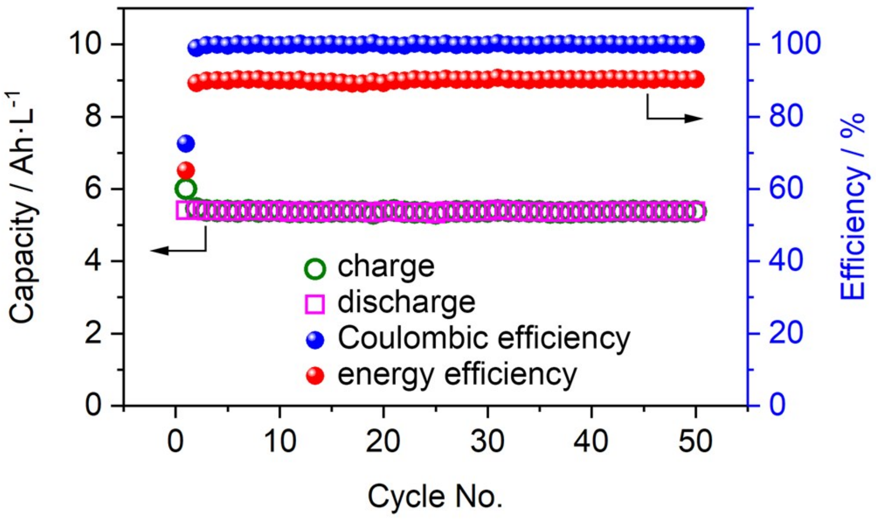


**Fig. S7** Cycling performance of Fe-tpy | Li half-cell.

**Table S2** A comparison of organic RFBs using viologen derivatives as anode materials

| Positive | Negative | Cell voltage [a] (V) | Aqueous or nonaqueous | Concentration [b] / solubility [c] | Energy density [d] (Wh/L) | Manuscript ref. No |
|---|---|---|---|---|---|---|
| 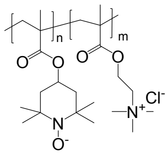 | 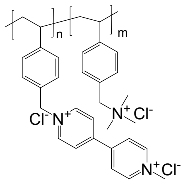 | 0.8–1.15 | aqueous | N/A | ca. 8.0 | 8e |
| 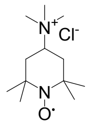 | 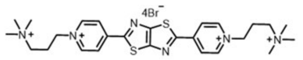 | 0.9–1.3 | aqueous | 0.1 M / 1.3 M in water or 1.1 M in 2 M NaCl | ca. 3.0 | 8g |
| 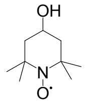 | 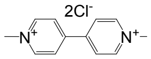 | 1–1.25 | aqueous | 0.5 M / 3M in water | ca. 1.8 | 13b |
| 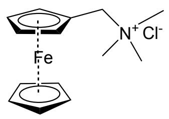 | 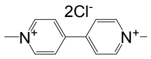 | 0.7–1 | aqueous | 0.5M / 3M in water | ca. 5.4 | 13c |
| 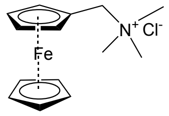 | 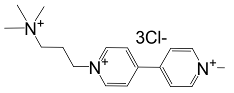 | 1.–1.3<br>0.5–0.8 | aqueous | 0.25M / 1.4 M in 2 M NaCl | ca. 4.2 | 13d |
| 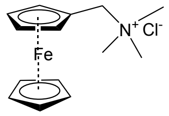 | 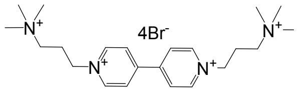 | 1–1.2<br>0.5–0.8 | aqueous | 0.25 M / 1.3 M in 2 M NaCl | ca. 4.1 | 13d |
| 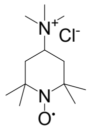 | 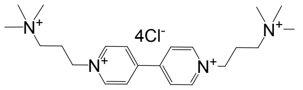 | 1.3–1.4<br>0.3–1.1 | aqueous | 0.05 M / N/A | ca. 2.3 | 13d |
| 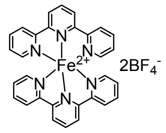 | 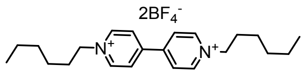 | 1.3–1.6 | non-aqueous | 0.25 M / 1.3 M in EC/DMC and 1.0 M in 1 M LiTFSI EC/DMC | ca. 4 | This work |

[a] refers to the discharge potential range

[b] refers to the concertation of the anolyte for cell performance evaluation

[c] refers to the solubility of anode materials

[d] refers to the achieved energy density calculated based on the total volume of the anolyte and catholyte

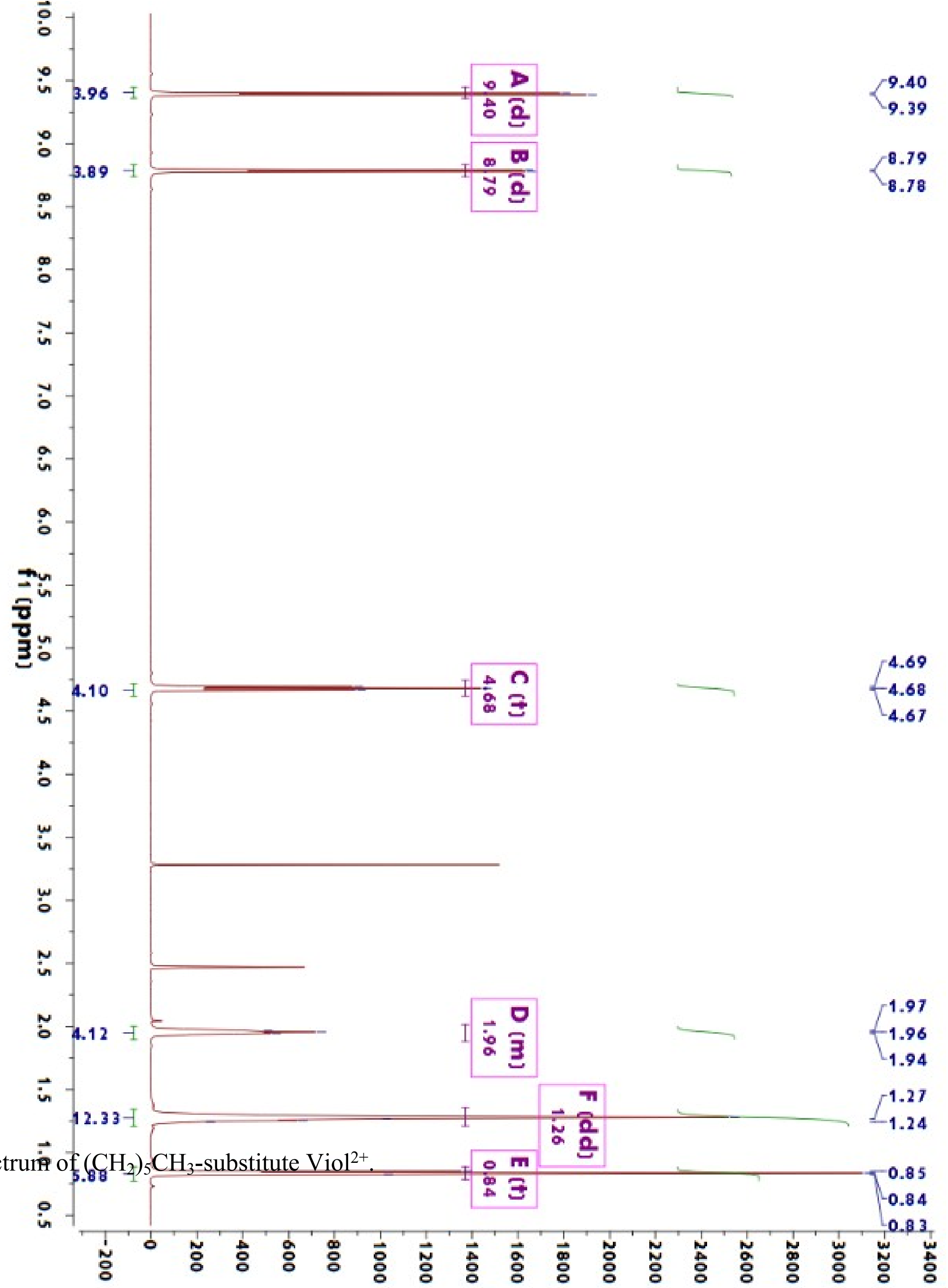


The $^{1}H$ NMR spectrum of $(CH_2)_5CH_3$-substitute $Viol^{2+}$.

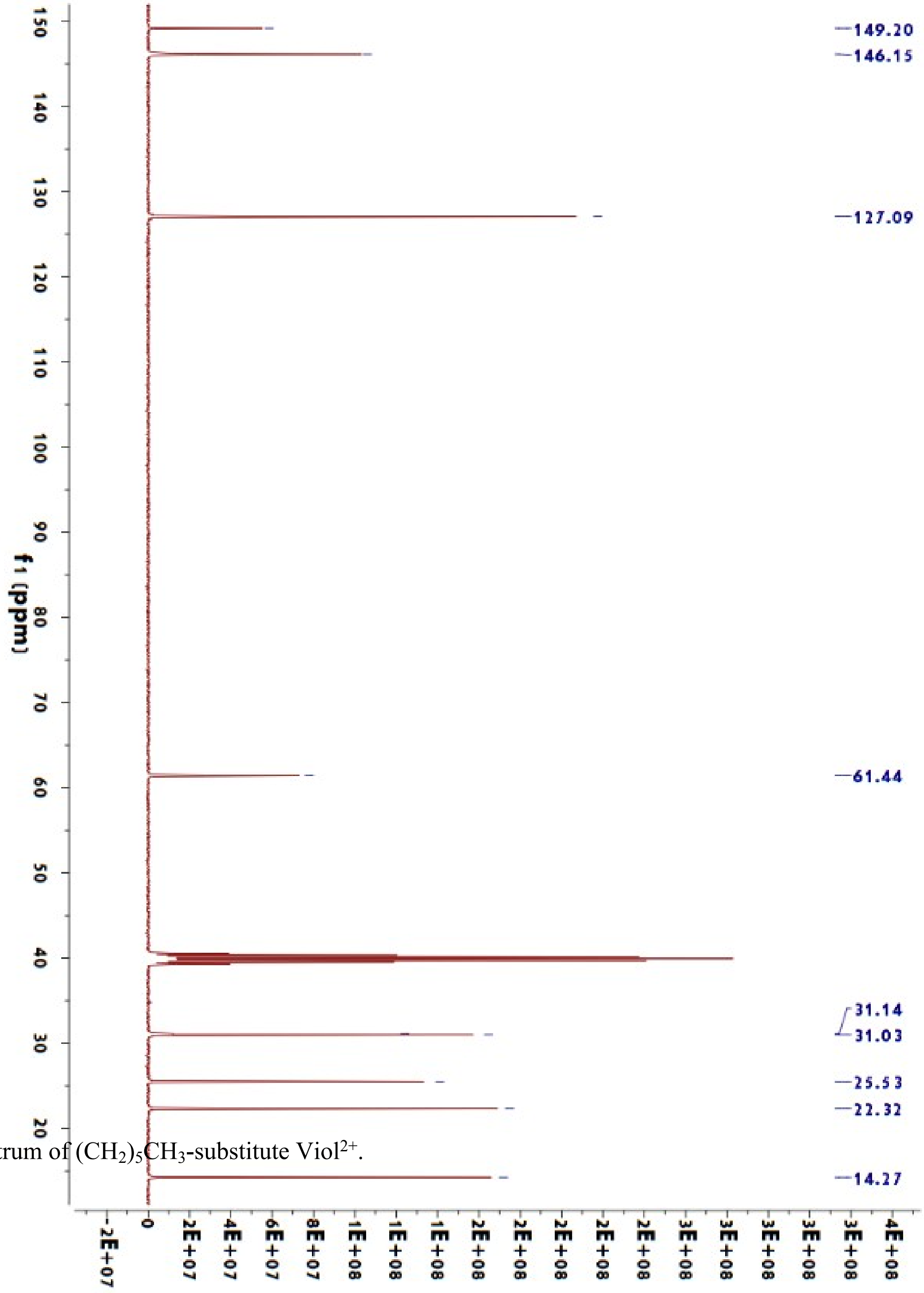


The $^{13}C$ NMR spectrum of $(CH_2)_5CH_3$-substitute $Viol^{2+}$.

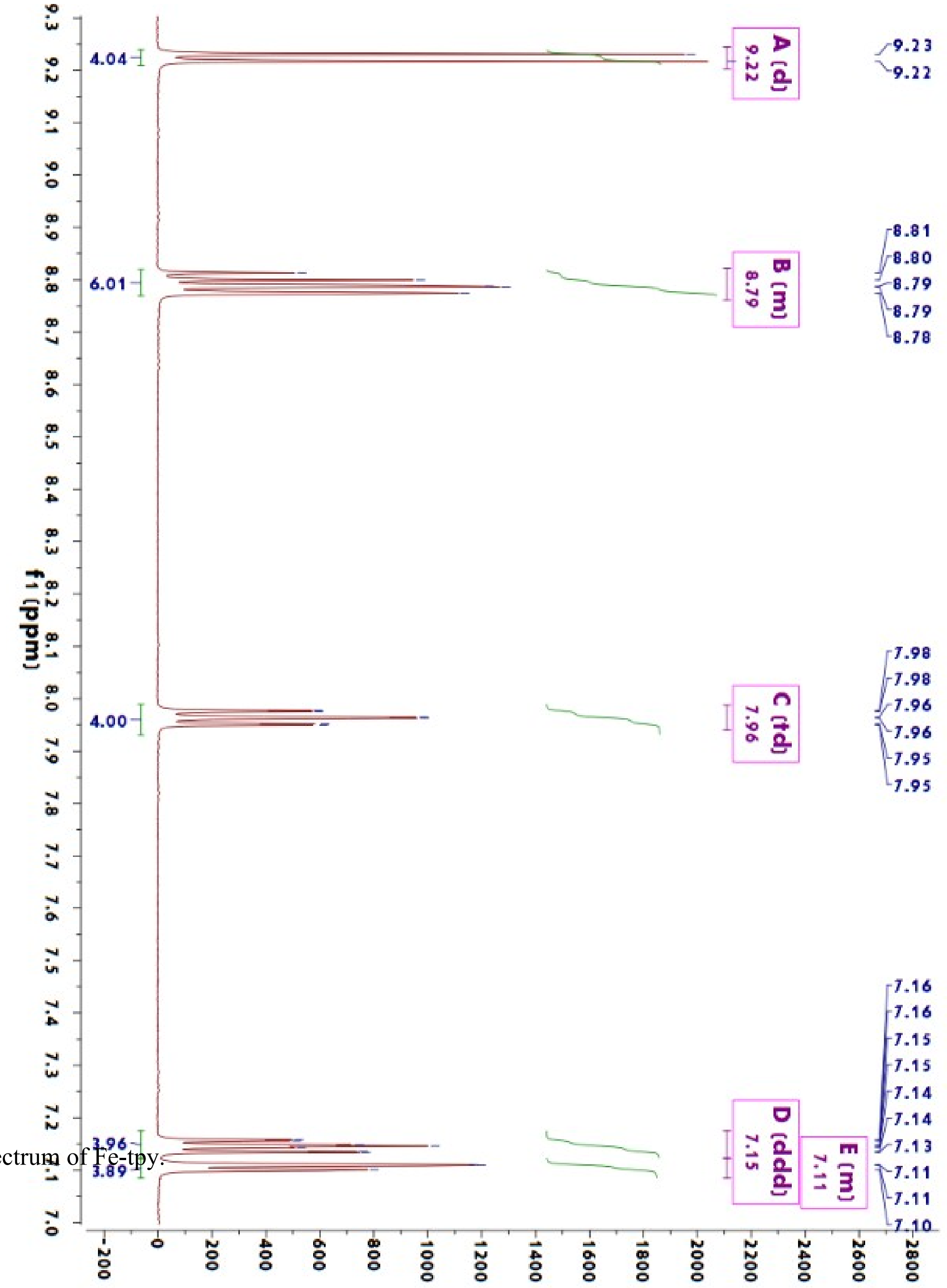


The $^{1}$H NMR spectrum of Fe-tpy.

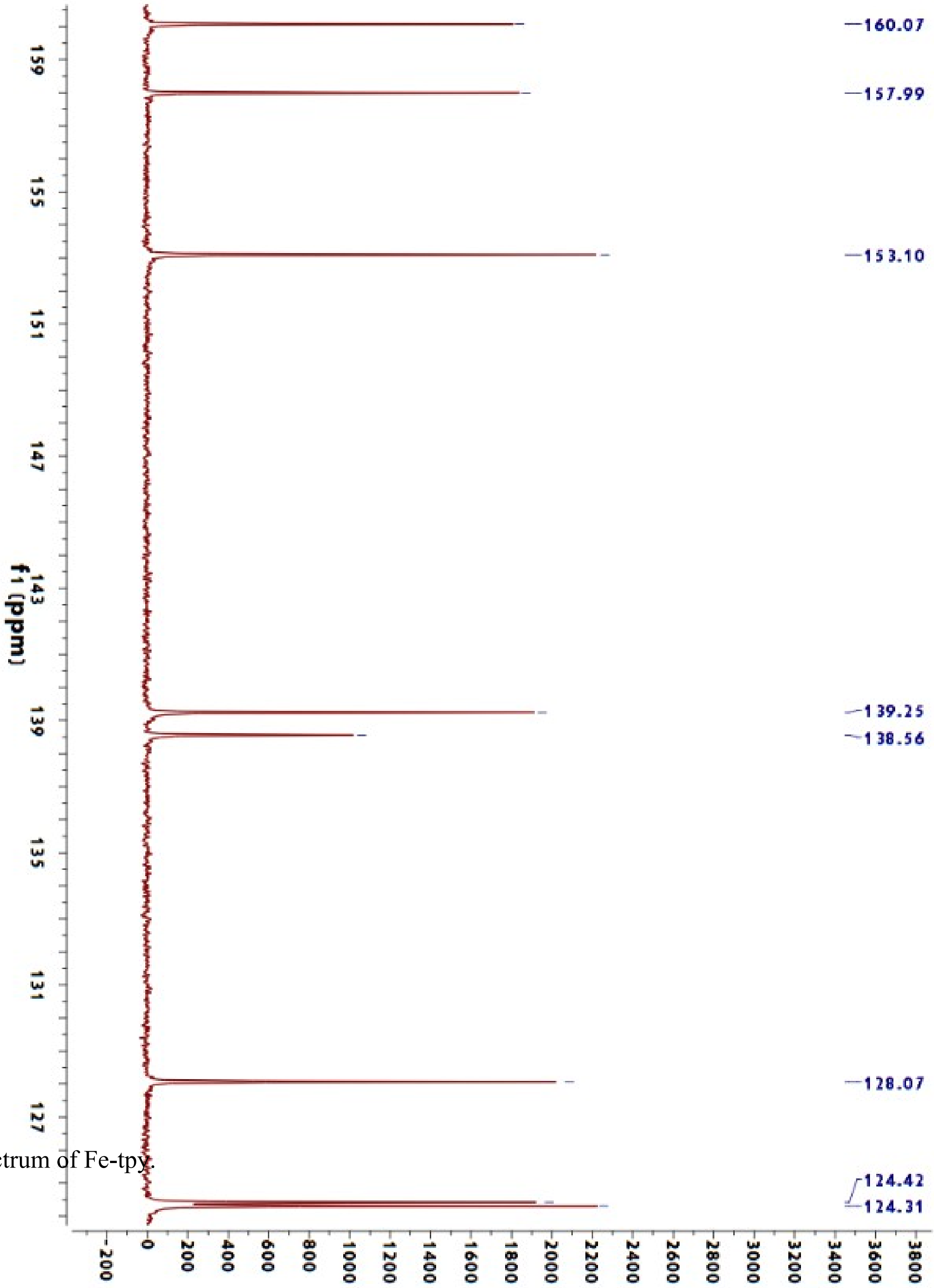


The $^{13}C$ NMR spectrum of Fe-tpy.

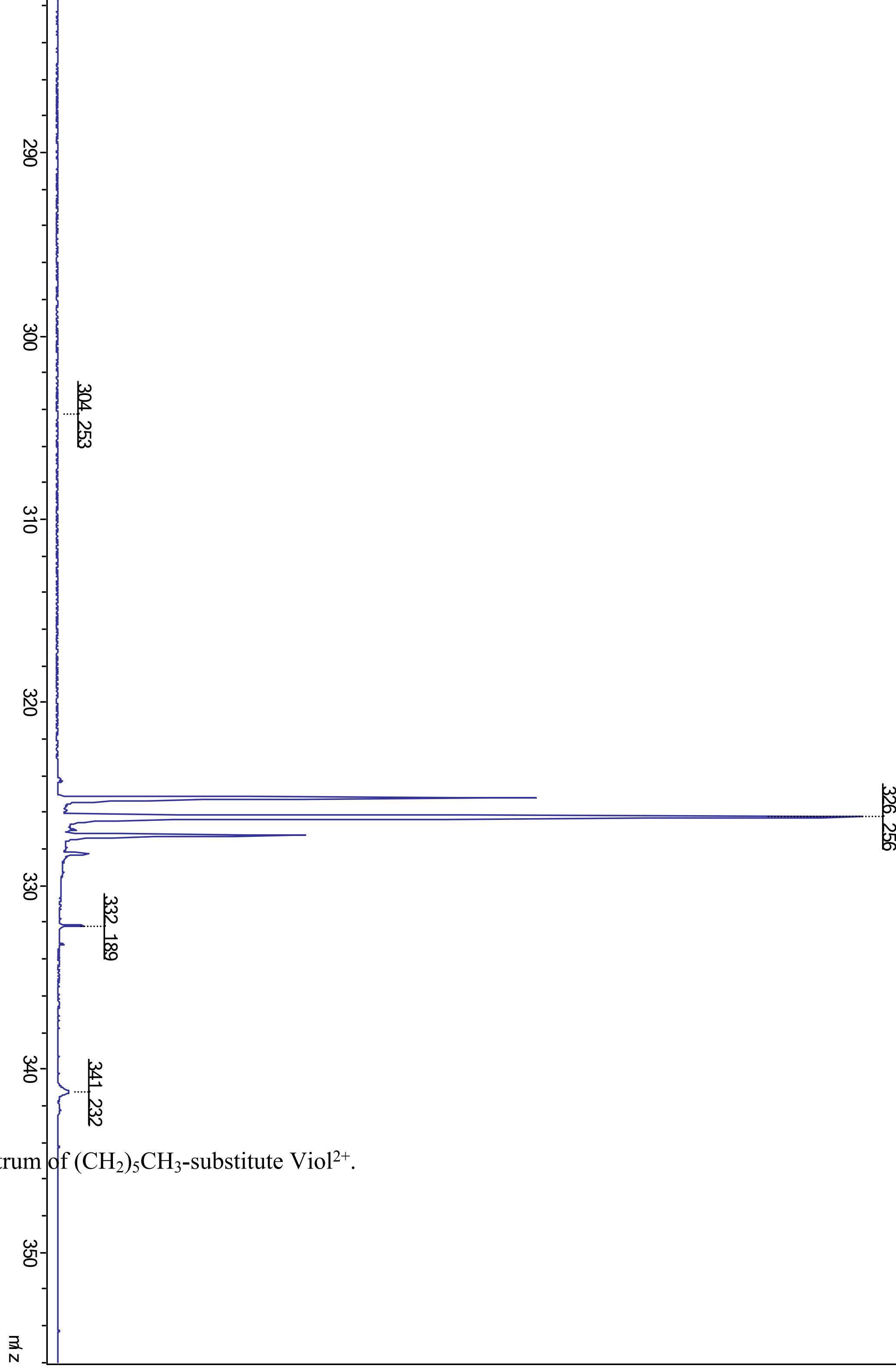


The MALDI-TOF-MS spectrum of $(CH_2)_5CH_3$-substitute $Viol^{2+}$.

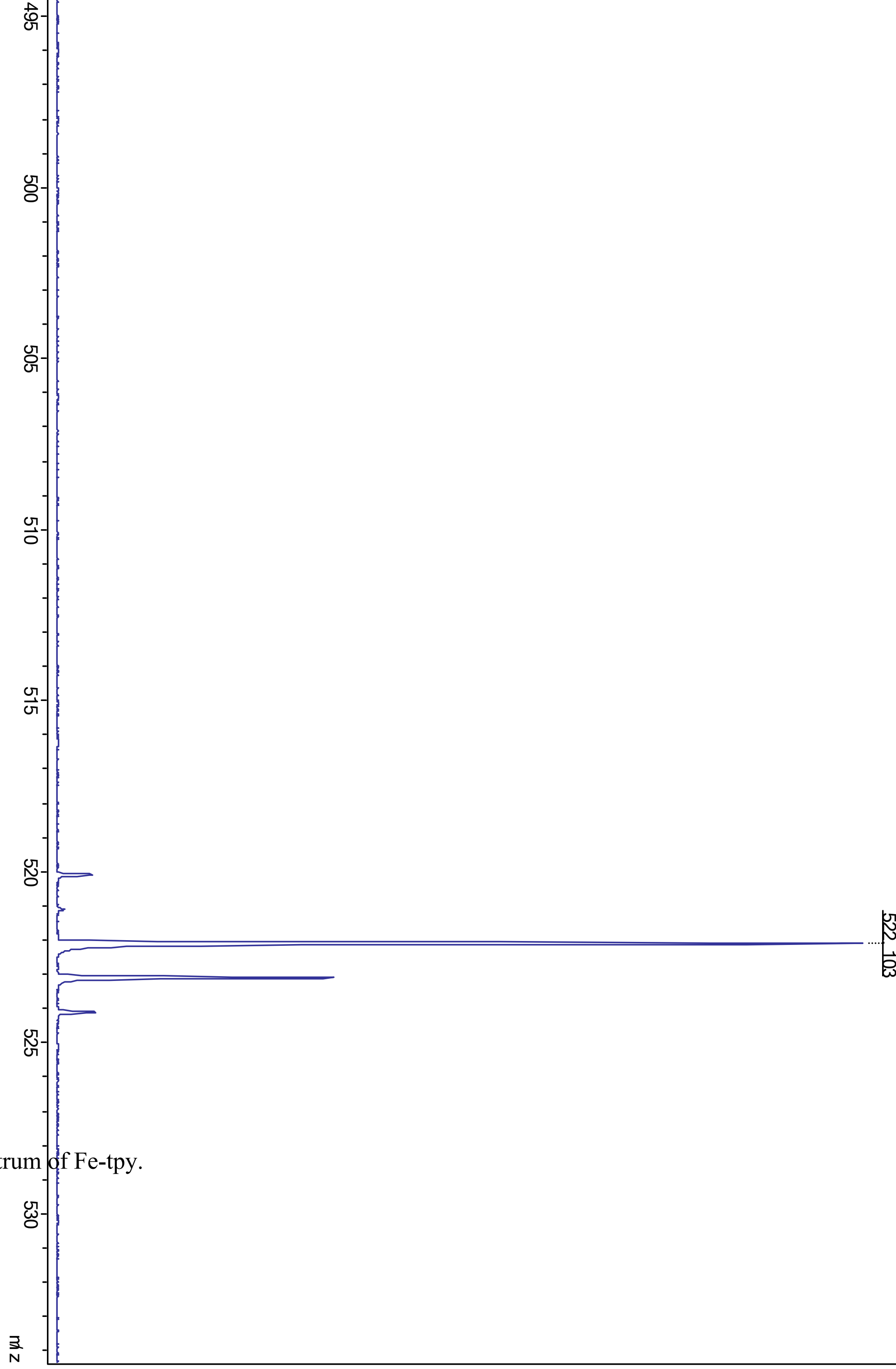


The MALDI-TOF-MS spectrum of Fe-tpy.
